# A Case of Severe Preeclampsia in the Last Trimester of Pregnancy Complicated by Acute Kidney Injury: A Case Report

**DOI:** 10.7759/cureus.115634

**Published:** 2026-09-02

**Authors:** Vasiliki Koutsia, Emmanouil M Xydias, Zoe Kotoula, Iliana Kotoula, Panagiotis Chouliaras, Konstantina Thymikiotou, Vasileios Migkakis, Georgios Toulios, Apostolos C Ziogas, Ioannis Thanasas

**Affiliations:** 1 Department of Obstetrics and Gynecology, General Hospital of Trikala, Trikala, GRC; 2 Department of Obstetrics and Gynecology, EmbryoClinic IVF, Thessaloniki, GRC; 3 Department of Anesthesiology, General Hospital of Trikala, Trikala, GRC; 4 Department of Surgery, General Hospital of Trikala, Trikala, GRC; 5 Department of Obstetrics and Gynecology, University of Thessaly, Larissa, GRC

**Keywords:** acute kidney injury, case report, diagnostic criteria, hemodialysis, preeclampsia, pregnancy, prognosis

## Abstract

Pregnancy-related acute kidney injury (AKI) secondary to severe preeclampsia is a rare but life-threatening obstetric complication for both the mother and the fetus-newborn. A 19-year-old primigravida at 36 weeks of gestation was admitted to the hospital with malaise and severe hypertension. Due to severe preeclampsia, delivery was completed by cesarean section. The diagnosis of AKI led to the initiation of hemodialysis, which was continued until full recovery of renal function. This case highlights the rare occurrence of AKI associated with severe preeclampsia during pregnancy and discusses the diagnostic approach and management of affected patients.

## Introduction

The older term “toxemia of pregnancy,” which referred to hypertension, proteinuria, and edema, has been replaced by the term “hypertensive disorders of pregnancy” [1]. The first reference to these disorders dates back approximately 5.000 years, when eclampsia (together with epilepsy) was among the first diseases recorded in human history [2]. More than a century has elapsed since the initial description of preeclampsia. Preeclampsia is a hypertensive disorder of pregnancy that typically occurs after the twentieth week of gestation and is associated with new-onset hypertension, usually accompanied by proteinuria and signs of end-organ dysfunction. In addition to proteinuria, end-organ involvement may include hepatic dysfunction, hemolysis, thrombocytopenia, seizures, stroke, and AKI [3].

AKI during pregnancy or the postpartum period is a rare but life-threatening obstetric complication for both the mother and the fetus/newborn. Hypertensive and thrombotic microangiopathic disorders have been recognized as major contributors to pregnancy-related AKI [4]. Although uncommon, its incidence has increased from 2.3 cases per 10,000 births in 1998 to 4.5 cases per 10,000 in 2008 [5]. The burden of pregnancy-related acute kidney injury remains higher in developing countries than in developed countries. Based on data from the World Health Organization, the maternal mortality ratio is 239/100,000 women in developing countries compared to 12/100,000 in developed countries. The burden of pregnancy-related AKI remains higher in developing than in developed countries [6].

This case report highlights the rare occurrence of AKI in a pregnant woman with preeclampsia with severe features during the third trimester, with particular emphasis on diagnostic criteria and management.

## Case presentation

A 19-year-old primigravida at 36 weeks of gestation (35 weeks and 4 days) presented to the Emergency Department of the General Hospital of Trikala, Greece, in a state of general malaise. The patient reported headache, dizziness, weakness, nausea, and vomiting. She had been followed regularly at the hospital’s obstetric outpatient clinic. The course of the pregnancy up to that point had been uneventful. There was no history of pre-existing hypertension prior to conception, nor of newly diagnosed hypertension before or after the 20th week of gestation. Gestational diabetes mellitus, diagnosed at the 27th week of pregnancy, was well controlled with dietary measures. The patient was a non-smoker. Body mass index was within the normal range (BMI = 23.7). No comorbidities were reported in her past medical history.

On physical examination, the patient appeared anxious and weak and exhibited edema of the lower extremities. She was afebrile (36.5 °C). Heart rate was 114 beats/min, and blood pressure was 165/110 mmHg, remaining elevated (>150/100 mmHg) on two consecutive measurements obtained over a 4-hour interval. Obstetric ultrasonography demonstrated normal fetal growth, a normal volume of amniotic fluid, and normal Doppler flow in the umbilical vessels. Hepatobiliary and renal ultrasonography of the pregnant woman revealed no abnormal findings. Cardiological evaluation, as well as neurological and ophthalmological examinations, were unremarkable. Immediately after blood sampling for urgent laboratory investigations (Table 1), the patient was admitted to the ward. Intravenous magnesium sulfate therapy was promptly initiated, with a loading dose of 5 g administered over 15 minutes, followed by continuous intravenous infusion at a rate of 2 g/hour for 24 hours. This was followed by slow intravenous administration of hydralazine at a dose of 5 mg over a period of 2 minutes. The dose of the drug was repeated 20 minutes later due to blood pressure remaining at levels >160/110 mmHg. Oliguria was noted (0.3 mL/kg body weight/hour). Urinalysis of a random urine sample revealed proteinuria (urinary protein +++). Protein excretion in the 24-hour urine collection was 2.15 g (laboratory reference value <300 mg).

**Table 1:** Hematological and biochemical tests that led to the diagnosis of severe preeclampsia-related acute kidney injury CS: Cesarean Section; Ht: Hematocrit; Hb: Hemoglobin; PLT: Platelets; WBC: White Blood Cells; NEUT: Neutrophils; APTT: Activated Partial Thromboplastin Time; INR: International Normalized Ratio; FIB: Fibrinogen; Glu: Glucose; Cr: Creatinine; AU: Acid Uric; Na+: Sodium; K+: Potassium; TBIL: Total Bilirubin; LDH: Lactate Dehydrogenase; SGOT: Serum Glutamic Oxaloacetic Transaminase; SGPT: Serum Glutamate Pyruvate Transaminase

Laboratory tests	Last obstetric visit	Entrance to the clinic	1^st^ postoperative day after CS	2^nd^ postoperative day after CS	4^th^ postoperative day after CS	Normal laboratory values
Ht	39.2%	38.3%	37.1%	36.4%	37.3%	37.7 – 49.7%
Hb	12.9 gr/dl	12.7 gr/dl	11.9 gr/dl	11.7 gr/dl	12.1 gr/dl	11.8 – 17.8 gr/dl
PLT	248x10^3^/ml	164x10^3^/ml	161x10^3^/ml	176x10^3^/ml	201x10^3^/ml	150 – 350 x10^3^/ml
WBC	9.44x10^3^/ml	10.35x10^3^/ml	16.38x10^3^/ml	12.51x10^3^/ml	7.29x10^3^/ml	4 – 10.8 x10^3^/ml
NEUT	75.6%	75.9%	85.9%	80.1%	67.1%	40 – 75%
aPPT	-	29.7	28.6	-	-	24.0 – 35.0 sec
INR	-	0.98	0.99	-	-	0.8 – 1.2
FIB	-	285 mg/dl	295 mg/dl	-	-	200 – 400 mg/dL
Glu	88 mg/dl	95 mg/dl	85 mg/dl	-	-	75 – 115 mg/dL
Cr	0.49 mg/dl	1.85 mg/dl	2.15 mg/dl	2.95 mg/dl	2.81 mg/dl	0.40 – 1.10 mg/dL
Uric Acid	3.4 mg/dl	7.3 mg/dl	7.1 mg/dl	6.9 mg/dl	6.2 mg/dl	2.4 – 6.0 mg/dL
Na^+^	-	141.9 mmol/L	139.7 mmol/L	-	-	136 – 145 mmol/L
K^+^	-	3.88 mmol/L	3.98 mmol/L	-	-	3.5 – 5.1mmol/L
TBIL	-	0.54 mg/dl	0.35 mg/dl	-	-	0 – 1.2 mg/dL
LDH	-	157 IU/L	144 IU/L	-	-	125 – 220 IU/L
SGOT	-	29 IU/L	28 IU/L	-	-	5 – 33 IU/L
SGPT	-	17 IU/L	15 IU/L	-	-	10 – 37 IU/L
Albumin	-	2.31 g/dL	2.29 g/dL	2.78 g/dL	3.01 g/dL	3.5 – 5.5 g/dL

New-onset hypertension during pregnancy accompanied by proteinuria, followed by a marked increase in serum creatinine levels, established the diagnosis of severe preeclampsia associated with AKI (Table 1). The haptoglobin value, reticulocytes, and schistocytes in the peripheral smear were normal. Immediate completion of delivery by cesarean section was therefore decided (Figure 1).

**Figure 1:**
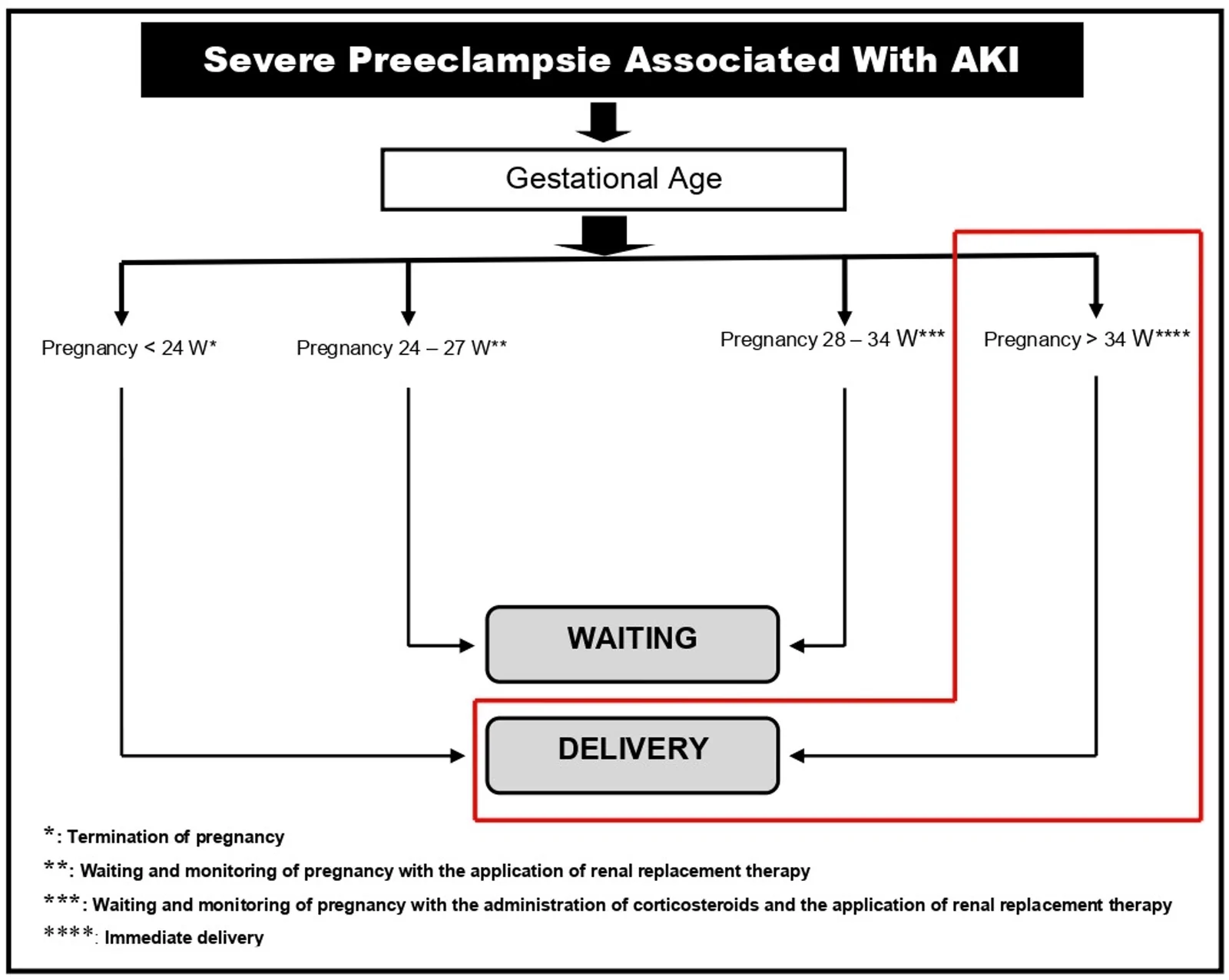
Flowchart of the management protocol for severe preeclampsia in our clinic Steps followed in the management of the present case (red outline) Image credit: The authors AKI: Acute Kidney Injury

The cesarean section was completed uneventfully, with normal intraoperative blood loss. A female neonate weighing 2.860 g was delivered, and admission to a neonatal intensive care unit was not deemed necessary. During the first postoperative days, maternal serum creatinine levels remained elevated (Table 1). A nephrological consultation led to the decision to initiate hemodialysis sessions. The decision to initiate hemodialysis was based on the increased and worsening creatinine value of >2 mg/dl and the increased risk of worsening our patient's respiratory condition. On the fifth postoperative day, the puerperal woman was discharged from the clinic together with her newborn. Hemodialysis was continued until complete recovery of renal function.

## Discussion

A diagnosis of AKI associated with severe preeclampsia requires the new onset of arterial hypertension after the twentieth week of gestation in combination with oliguria (<0.5 mL/kg body weight/hour lasting 6-12 hours), proteinuria, neurological or visual disturbances, epigastric or right upper quadrant pain, and fetal manifestations such as intrauterine growth restriction. The presence of proteinuria of >2 g in pregnant women with a systolic blood pressure of ≥160 mmHg and/or a diastolic blood pressure of ≥110 mmHg on at least 2 measurements obtained at least 4 hours apart, along with normal blood pressure prior to conception, establishes the diagnosis of severe preeclampsia and necessitates careful monitoring of maternal renal function [7].

Monitoring of maternal renal function is based on the assessment of serum creatinine levels. It is estimated that in a normally progressing pregnancy, the glomerular filtration rate increases by 40%-60%, resulting in a physiological reduction in plasma creatinine levels in pregnant women (reference range during pregnancy: 0.4-0.6 mg/dL) [8]. Therefore, an increase in serum creatinine of >0.3 mg/dL from baseline or >1.5 times the reference value constitutes an important criterion for the diagnosis of AKI in pregnancy [9]. In addition, in patients with severe preeclampsia, elevated plasma uric acid levels are significantly associated with an increased risk of developing AKI [7]. In our patient, severe hypertension (165/110 mmHg) followed by marked proteinuria (2.15 g) and a significant rise in serum creatinine (1.85 mg/dL) established the diagnosis of AKI associated with severe preeclampsia, based on which a timely and effective management plan was implemented for this rare obstetric complication.

The management of AKI during pregnancy involves systematic monitoring of the mother, continuous assessment of fetal status, and administration of appropriate therapeutic interventions [10]. In cases of severe preeclampsia, depending on gestational age, pregnancy termination may be required; close monitoring with renal replacement therapy may be implemented; or immediate completion of delivery may be performed via vaginal birth or cesarean section (Figure 1). Renal replacement therapy in pregnant women is indicated in cases of worsening renal function and when awaiting delivery. Although timely and safe delivery of the fetus should remain a priority, hemodialysis has been shown to significantly improve perinatal outcomes in pregnant women with AKI [11]. Additionally, a case reported in the literature described severe preeclampsia associated with AKI in which administration of eculizumab (a humanized IgG monoclonal antibody targeting complement protein C5) led to significant improvement in hematologic, hepatic, and renal function, ultimately allowing discontinuation of renal replacement therapy [12]. In our patient, there was no dilemma regarding continuation or monitoring of the pregnancy. The advanced gestational age (35 weeks and 4 days) led to the decision to perform a cesarean section, thereby avoiding the risk of additional maternal and fetal complications that could adversely affect prognosis.

Prognosis is generally poor, particularly in patients who are not managed appropriately and in a timely manner. Pregnancy-related AKI represents a global health concern associated with a high risk of maternal and perinatal morbidity and mortality [10]. In developing countries, maternal mortality related to pregnancy-associated AKI increases with the severity of complications and may range from 13% to 24% [13].

## Conclusions

In conclusion, this rare case underscores the importance of careful monitoring of renal function in pregnant women with preeclampsia. It also highlights the importance of early recognition, monitoring of renal function, and multidisciplinary management.
